# Warming affects routine swimming activity and novel odour response in larval zebrafish

**DOI:** 10.1038/s41598-023-48287-y

**Published:** 2023-11-29

**Authors:** Jade M. Sourisse, Lucrezia C. Bonzi, Julie Semmelhack, Celia Schunter

**Affiliations:** 1 The Swire Institute of Marine Science, School of Biological Sciences, The University of Hong Kong, Pokfulam Road, Hong Kong, Hong Kong SAR China; 2 The Division of Life Science, Department of Chemical and Biological Engineering, The Hong Kong University of Science and Technology, Clearwater Bay, Kowloon, Hong Kong SAR China

**Keywords:** Climate-change ecology, Freshwater ecology, Molecular ecology

## Abstract

Temperature is a primary factor affecting the physiology of ectothermic animals and global warming of water bodies may therefore impact aquatic life. Understanding the effects of near-future predicted temperature changes on the behaviour and underlying molecular mechanisms of aquatic animals is of particular importance, since behaviour mediates survival. In this study, we investigate the effects of developmental temperature on locomotory behaviour and olfactory learning in the zebrafish, *Danio rerio*. We exposed zebrafish from embryonic stage to either control (28 °C) or elevated temperature (30 °C) for seven days. Overall, warming reduced routine swimming activity and caused upregulation of metabolism and neuron development genes. When exposed to olfactory cues, namely catfish cue, a non-alarming but novel odour, and conspecifics alarming cue, warming differently affected the larvae response to the two cues. An increase in locomotory activity and a large transcriptional reprogramming was observed at elevated temperature in response to novel odour, with upregulation of cell signalling, neuron development and neuron functioning genes. As this response was coupled with the downregulation of genes involved in protein translation and ATP metabolism, novel odour recognition in future-predicted thermal conditions would require energetic trade-offs between expensive baseline processes and responsive functions. To evaluate their learning abilities at both temperatures, larvae were conditioned with a mixture of conspecifics alarm cue and catfish cue. Regardless of temperature, no behavioural nor gene expression changes were detected, reinforcing our findings that warming mainly affects zebrafish molecular response to novel odours. Overall, our results show that future thermal conditions will likely impact developing stages, causing trade-offs following novel olfactory detection in the environment.

## Introduction

Olfaction is one of the main ways fish gather information about their environment. A wide variety of behaviours is influenced by the detection of olfactory stimuli, such as feeding, migration, reproduction and predator escape response^1^. One famous example is the accidental discovery of fear reaction to alarm cues in fish: when a minnow (*Phoxinus phoxinus*) is injured, an alarm cue is released and nearby conspecifics drastically change their behaviour by swimming away and moving in tighter schools^2^. Such olfactory-mediated behaviours are common in fish^3^ and positively affect survival not only in adults but also in immature life stages, and many fish larvae can assess risks by sensing cues from predators or other prey^4^. While some of the olfactory processes important for survival are innate, others involve associative learning: if during a conditioning phase two odours occur together, later in life the second odour will be associated with the same events and situations as the first odour^5^. This is the case with predator recognition, which has been described in the European minnow. After sensing the odour of a natural predator mixed with alarm cues released by the skin of conspecifics, minnows would start hiding and schooling upon smelling the pike’s odour alone^6^. Such an association of odours causing antipredator behaviours to otherwise neutral stimuli occurs in a diverse range of prey fishes^7^, notably zebrafish^8^.

Environmental factors have the potential to influence olfactory responses^9^, with temperature arguably one of the main environmental variables influencing the performance of fish^10,11^ including effects on olfactory triggered feeding^12^. Global warming, which is happening at an unprecedented rate in aquatic environments^13,14^, may therefore alter crucial processes in fish, including olfactory and behavioural responses. Olfaction in fish is mediated by olfactory sensory neurons that converge on the olfactory bulb, where there is an exchange of information to second-order neurons, that are likely involved in the learning process^15^. Physiological changes in the olfactory bulb may occur due to temperature^16,17^ and warming can impair fish associative learning due to alterations in memory formation^18,19^. Despite previous evidence of temperature altering gene expression involved in olfaction^20^, it is unclear to what extent or how near-future thermal conditions will impact the sensory system and overall cue processing in fishes^21^, particularly at the molecular level. For this reason, understanding how warming modulates the behaviour and learning experiences that fish need for survival is of crucial importance, especially in light of rapid climate change.

In our study, we investigate the effects of developmental temperature on the locomotory behavioural and genome-wide transcriptomic responses of zebrafish larvae to two different olfactory cues: an alarm substance (Conspecifics Alarm Cue) and a non-alarming odour (catfish cue). We hypothesize that future predicted thermal conditions for the end of the century will impact the molecular state in sensory processing during the behavioural response to different cues. By performing classical conditioning, we assess whether a climate change-driven increase in temperature influences the olfactory learning experience, both at behavioural and molecular levels. Overall, by characterizing temperature-specific whole body transcriptomic and behavioural changes in response to olfactory cues, we aim to determine how future predicted temperature will affect the environmental perception of zebrafish and their learning experience through olfaction.

## Methods

### Animals, housing and temperature exposure

Wild-type breeders (nine females, ten males; AB strain) were obtained from the Hong Kong University of Science and Technology zebrafish husbandry facilities. Wild-type zebrafish were kept at 28 °C and housed in two recirculating systems (80 × 37 × 32 cm) with six males/four females in one and four males/six females in the other, with a 14/10 h light–dark cycle. The thermal conditions were measured, adjusted and recorded every 60 s with heaters (Schego) and a STC-1000 Thermostat (Elitech). Fish were fed twice a day with TetraMin pellets. pH and nitrate levels were measured weekly using a WP-91 pH meter (TPS) and a HI97728 nitrate photometer (Hanna Instruments), respectively. Fertilized eggs (at the cleavage period of development^22^) were collected in the morning and then placed in Petri dishes filled with Danieau’s solution embryo medium (17 mM NaCl, 2 mM KCl, 0.12 mM MgSO_4_, 1.8 mM, Ca(NO_3_)_2_, 1.5 mM HEPES) in DSI-060D incubators (Digisystem) at either control (28 °C) or elevated (30 °C) temperature. Temperature inside the incubators was adjusted every 60 s. We selected this control temperature as it is the optimal rearing temperature of zebrafish in laboratory settings and chose the treatment temperature as + 2 °C as current IPCC scenarios estimate that because of climate change temperatures will globally increase between 1.5 °C and 2 °C above pre-industrial levels by the end of the century^13^. The embryo medium was changed daily. Embryos were reared under a 14/10 h light–dark cycle and from five days post fertilization (dpf) onwards they were fed a larval diet (Zeigler Bros) daily until seven dpf, which is a critical life stage^23^ during which larvae become active in their environment^22^ and can detect olfactory information with a functional sensory system^24^. Temperatures between treatments differed significantly (Wilcoxon rank sum test, p-value < 0.001) and did not follow a normal distribution (Shapiro–Wilk test, p-value < 0.001): the “treatment” temperature over the experimentation period was 30.1 ± 0.3 °C, whereas the mean control temperature was 28.2 ± 0.3 °C (SFig. 1). This study was carried out in approval of the Committee on the Use of Live Animals in Teaching and Research (CULATR) of the University of Hong Kong (# 5504-20), all methods were performed in accordance with the CULATR guidelines and regulations as well as the ARRIVE guidelines.

### Behavioural assays and analyses

**Figure 1:**
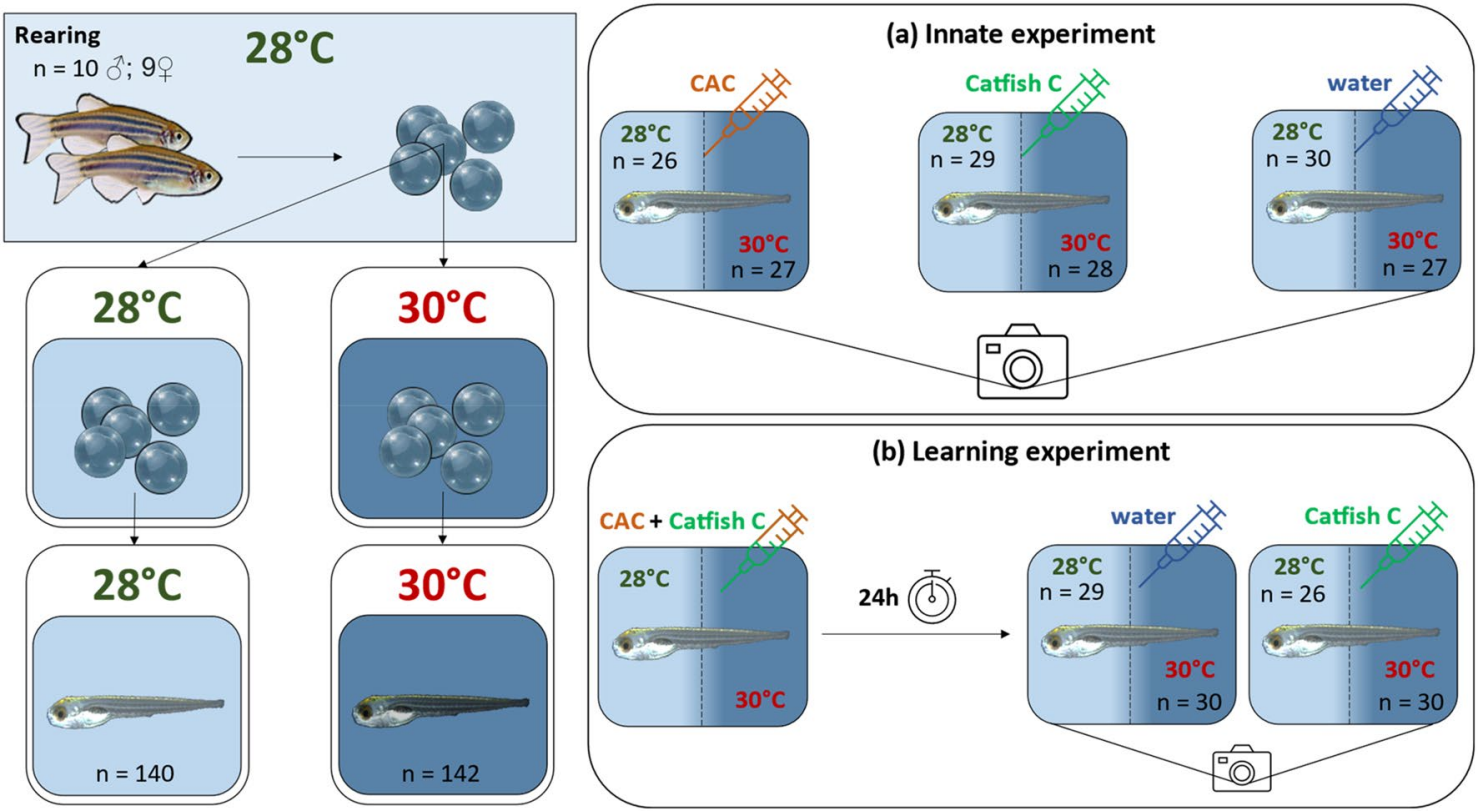
Experimental designs of the (**a**) innate and (**b**) learning experiments; “n” values correspond to the number of individuals within each group; treatments are illustrated by a syringe which colour corresponds to the specific cue the larvae are exposed to (Conspecifics Alarm Cue, CAC, or catfish cue, Catfish C, or water control); in the learning experiment, conditioning consists in exposing the larvae to a mixture of CAC and Catfish C.

To test the innate response of zebrafish larvae to olfactory cues, we conducted a first experiment referred to as “the innate experiment”. Larvae were exposed to Conspecifics Alarm Cue (CAC), a substance that is known to trigger an alarm response such as reduced locomotion or increased freezing, which consists in a total suppression of the swimming activity^8,25^. The catfish cue (Catfish C) was chosen to assess the larval response to the odour of a fish species that zebrafish do not innately fear, as used in previous research^26^. Because lab-reared zebrafish were not previously exposed to catfish or Catfish C, catfish odour would not elicit anti-predation responses, since recognition of this species by zebrafish would only occur from associative learning. The CAC was produced by sacrificing zebrafish larvae reared together with the experiment subjects by head concussion, and homogenizing their bodies in water at a concentration of one donor larva/mL with a sterile mortar and pestle, 5 min before cue exposure time^26^. For Catfish C, water in which two shark catfish fed with Algae wafers (Hikari) were maintained for 24 h (*Pangasianodon hypophthalmus*; length:water ratio of 20 cm:7L) was used, following the methodology by Lucon-Xiccato et al.^26^. Seven dpf larvae were transferred to experimental acrylic chambers (40 × 40 × 9 mm each) with a transparent bottom filled with six mL of Danieau’s solution that had been maintained at the corresponding larval rearing temperature and gradually reached a common room temperature of 26 °C during an acclimation period. Larvae were given 40 min acclimation time, then recorded from above using a Canon EOS M50 camera. After a baseline observation period of 12 min, larvae were exposed to either CAC (n = 26 for control temperature, n = 27 for treatment temperature), Catfish C (n = 29 for control temperature, n = 28 for treatment temperature) or control water (n = 30 for control temperature, n = 27 for treatment temperature) for another 12 min (Fig. 1a). Each cue was introduced in the chamber at a volume of 0.5 mL, using a 2.5 mL syringe.

To test whether seven dpf zebrafish can learn to associate the non-alarming catfish cue with a dangerous signal and whether temperature has an influence on that response, we conducted a second experiment referred to as “the learning experiment”. Larvae were introduced at six dpf to the experimental acrylic chambers, acclimated for 40 min and then conditioned to 1 mL of a mixture of CAC (0.5 mL) and Catfish C (0.5 mL), prepared as described above. The conditioning phase lasted 40 min, then the larvae were placed back into the incubation chambers for 24 h, following the methodology by Lucon-Xiccato et al.^26^. On the following day, larvae were placed back in individual chambers, where they were given 40 min to acclimate and were then video recorded. After a baseline activity period of 12 min, larvae were exposed to either control water (n = 29 at control temperature; n = 30 at elevated temperature) or Catfish C alone (n = 26 at control temperature; n = 30 at elevated temperature) for another 12 min, as in experiment 1 (Fig. 1b). Larvae from both experiments were immediately snap frozen in liquid nitrogen after the behavioural assays and stored at − 80 °C for further processing.

To standardize the length of all videos to 12 min, raw videos were cut in Adobe Premiere 2019. The videos were then processed in DeepLabCut v 2.2.0^27^ for body coordinates tracking of multiple animals per video. For a training dataset for machine learning of a network (Max. it. = 50 000; Saved it. = 5000; kept snapshots = 10) body parts were manually labelled in 20 frames per video among a representative set of 10 videos across dates and treatment. Incorrect tracklets were manually corrected post-analysis in DeepLabCut and positions through time of each larva were extracted in R v 4.2.1^28^ using the trajr package v 1.4.0^29^. Trajectories in which position was retrieved in less than 80% of all frames were discarded (Table S1). With the filtered data, whole distances travelled for each larvae every minute in each video were calculated using body coordinate values across frames and the trajr R package v 1.4.0^29^ (Table S2). Finally, this value was statistically compared in R for each larva: the values before exposure to the cues were paired to those after exposure. The paired values were statistically compared using a paired t-test when the distribution was normal, or else with a Wilcoxon signed-rank test, to assess whether the cue influenced the behaviour. Finally, the length of each larva was measured using the software JMicroVision v 1.3.4^30^: a random frame of each raw video per experiment was extracted and the one-dimensional scale was set to 40 mm of the behavioural chamber. Whole distance travelled for each larvae through time could then be expressed in “body length” unit, dividing distances (mm) by each larva’s body length (mm), to account for body length potentially driving differences in swimming performance.

### RNA sequencing and gene expression analyses

In order to assess the broad transcriptomic response of zebrafish to olfactory cues and the potential influence of temperature, total RNA was extracted from whole larvae (n = 10 per treatment) using a TRIzol™ Plus RNA Purification Kit with Phasemaker tubes (Thermo Fisher) due to the small size of the larvae. RNA concentration was measured using Qubit™ RNA HS Assay Kit (Thermo Fisher Scientific) and quality assessed using TapeStation (Agilent). RNA was sequenced at 150 bp paired end on an Illumina NovaSeq at the Centre for PanorOmic Sciences (CPOS) of the University of Hong Kong. After sequencing, raw sequence data (on average 32,022,942 ± 3,345,742; Table S3) were trimmed for adapters and filtered based on read quality using Trimmomatic^31^ with the following parameters: “ILLUMINACLIP: all_adapters.fa:2:30:10:8:TRUE LEADING:4 TRAILING:3 SLIDINGWINDOW:4:20 MINLEN:36”. High quality reads (on average 29,879,111 ± 3,122,986; Table S3) were then mapped against the reference genome (Genome Reference Consortium z11) from Ensembl^32^, but using the more comprehensive zebrafish transcriptome annotation from Lawson et al.^33^. Mapping was done using the program HISAT2^34^ to obtain expression levels across the genome and 15,691 genes out of 36,351 mapped genes were associated to GO terms in the Lawson annotation of the GRCz11 zebrafish genome (Table S4). For functional enrichment analysis, the functional annotation file retrieved from the Lawson lab website that associated the gene IDs with ZFIN IDs^35^, Ensembl IDs and/or Entrez IDs^33^, and from a custom reference set file created using BioMart resources (containing Ensembl IDs, ZFIN IDs and Entrez IDs) and the R package “FindMyFriends”^36^.

Finally, differential expression analyses were conducted using DESeq2 v 1.38^37^ to investigate which genes are differentially expressed (DE) between control larvae and treatment larvae between samples from the same experiment (innate or learning). The larvae’s date of fertilization (df) was found to be an important factor influencing the differential expression of genes, so it was kept in the statistical model of both experiments (Likelihood Ratio Test, “df” accounted for respectively 14.3% and 51.8% of the DE genes in experiment 1 and 2). The formula contained a combination of the two factors (temperature and cue) with therefore six possible levels, allowing to make specific pairwise comparisons between the most specific groups of the experimental design (“ ~ df + combination”). Differentially expressed genes with a baseMean under 10 and/or an absolute value of log2foldchange inferior to 0.3 were discarded to ensure that differential expression was not an artifact of low counts, and to increase stringency.

For the significantly differentially expressed (DE) genes, functional enrichment analyses were performed in OmicsBox v 1.4.11 (Fisher’s Exact Test). The GO terms get associated with the gene IDs because of their correspondence with either one of the three other types of IDs: if there was no documented Ensembl ID, the Entrez ID would be used and if the two former ones were absent, then the ZFIN ID would be used. The Gene Ontology (GO) terms with an FDR adjusted p-value^38^ below the 0.05 threshold were considered enriched and reduced to most specific.

## Results

### Behavioural response to olfactory cues

**Figure 2:**
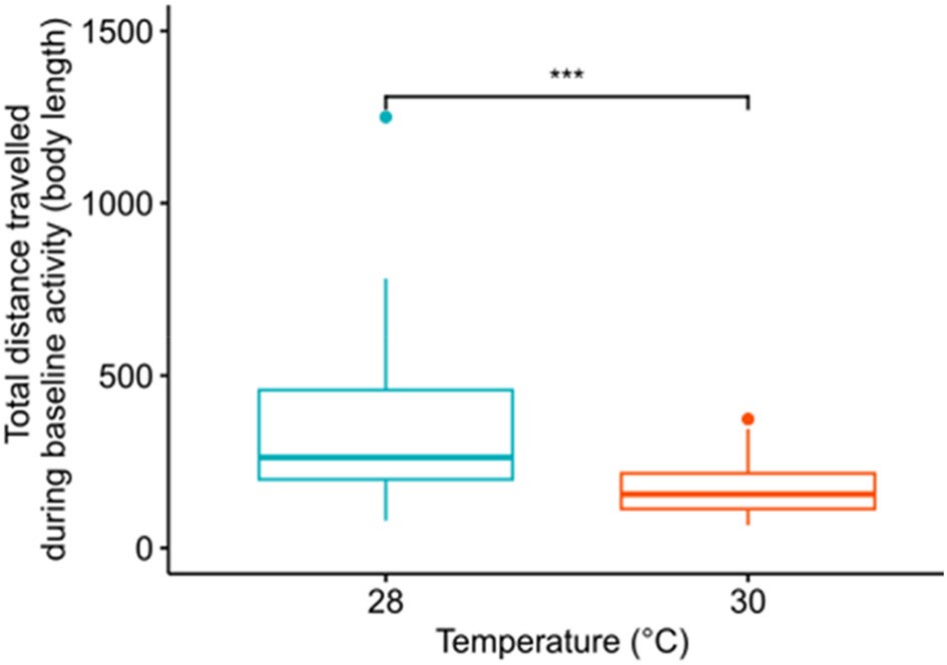
Effect of elevated temperature on routine swimming activity as total distance travelled by the larvae of the innate experiment during their baseline activity period in control (28 °C; green) or treatment (30 °C; red); dots represent outliers and one outlier of the control group was removed (distance = 4000 mm); stars (***) indicate the significant difference between mean values.

We hypothesized that warming would alter the swimming behaviour of zebrafish larvae. There was a significant effect of rearing temperature on baseline swimming distance in the innate experiment (Wilcoxon test, p-value < 0.001), with larvae swimming on average 332.4 ± 202.3 body lengths if reared in control temperature (n = 67) but only 176.2 ± 78.9 body lengths when reared at elevated temperature (n = 62), during the baseline activity period (Table S2 & Fig. 2). Although one individual reared at control temperature travelled more distance than the others from the same group, the observed difference was still significant in its absence. Similarly, rearing temperature had a significant effect on baseline swimming distance in the learning experiment involving conditioned larvae (Wilcoxon test, p-value < 0.005), with larvae swimming on average 368.6 ± 220.9 body lengths when reared at control temperature (n = 50), but only 258.3 ± 183.8 body lengths if reared at elevated temperature (n = 47; Table S2 & Fig. 2). This provides two independent measures in separate experiments revealing an effect of elevated temperature on the routine swimming distance of zebrafish larvae.

Overall, there was no significant innate effect of any olfactory cue exposure on swimming activity at control temperature (Tables S1 and S2, SFig 2). Although not significant, there was a slight increase in the average distance travelled after exposure to any cue in the innate experiment, due to individuals showing variable swimming activity. In control thermal conditions, control water did not affect the total distance travelled by naive larvae, as expected, with larvae swimming 357.7 ± 251.8 body lengths during baseline period and 368.1 ± 243.2 body lengths once exposed to control water (n = 24, Wilcoxon test, p p-value = 0.814). Exposure to catfish cue did not reduce the total distance travelled either (n = 24, Wilcoxon test, p-value = 0.310; baseline distance = 302 ± 164.7 body lengths, exposure distance = 361 ± 203.1 body lengths), nor did exposure to CAC (n = 19, paired t-test, p-value = 0.571; baseline distance = 338.9 ± 179.5 body lengths, exposure distance = 353.3 ± 185.5 body lengths).

**Figure 3:**
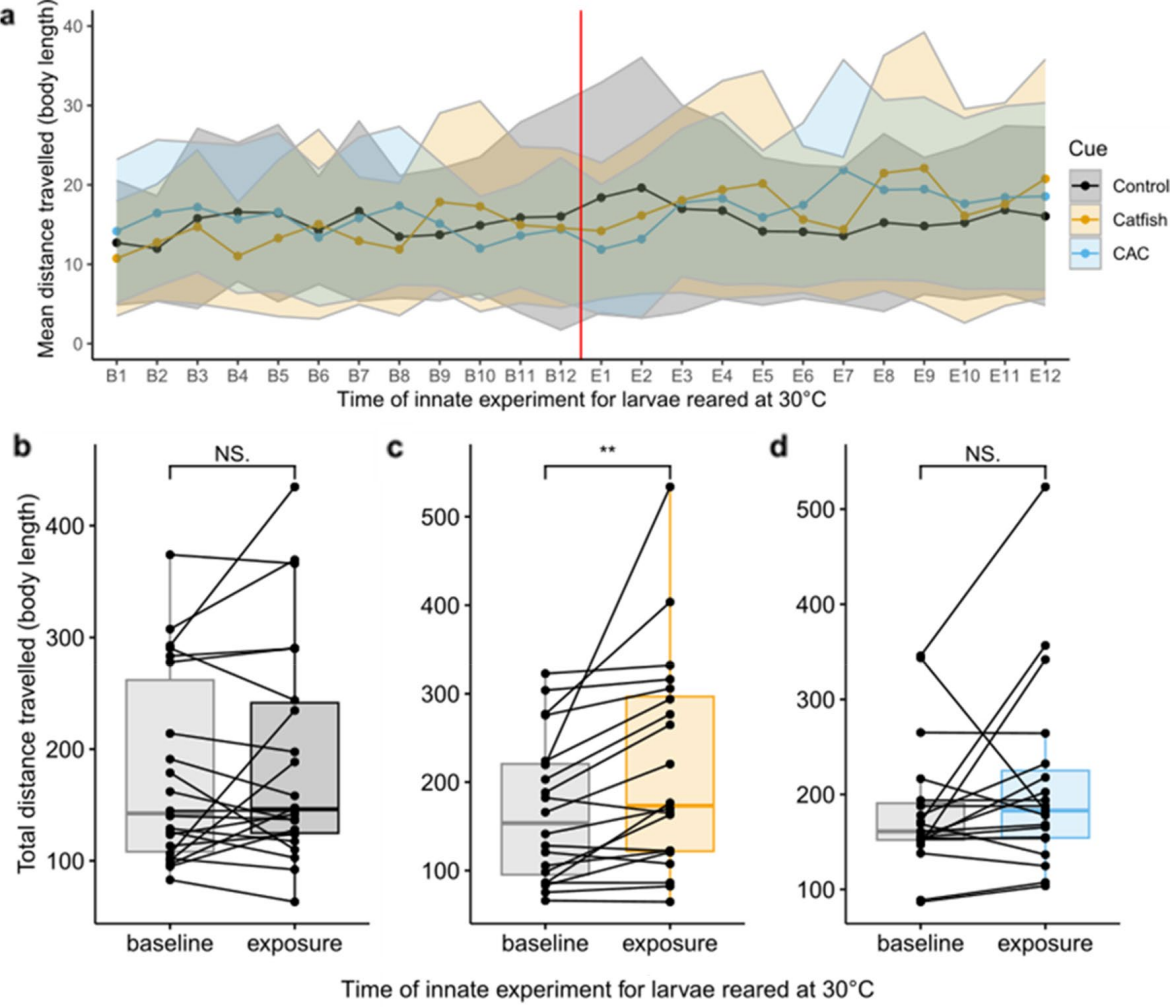
Effect of elevated temperature on swimming response to olfactory cue exposure as mean distance per minute (**a**) and total distance (**b**–**d**) travelled by larvae reared at elevated temperature, before cue exposure (B; baseline) and after cue exposure (E; exposure) for each cue group: control water (C, black), catfish cue (Catfish C, orange) or Conspecifics Alarm Cue (CAC, blue); ribbons around solid lines correspond to standard deviation; black dots linked by full lines represent paired individual comparisons of before and after the cue exposure; stars (**) indicate a statistical difference between mean values whereas “NS.” stands for “non-significant”.

At elevated temperature, exposure to control water did not affect the total distance travelled by naive larvae (n = 22, Wilcoxon test, p-value = 0.606; Fig. 3a and b). Larvae exposed to control water travelled on average 178.7 ± 86.8 body lengths during the baseline period and 191.7 ± 101 body lengths during the exposure period. Exposure to catfish cue, on the contrary, significantly increased the total distance travelled at elevated temperature (n = 20, paired t-test, p-value = 8.068 × 10^–3^; baseline distance = 167.6 ± 81.6 body lengths, exposure distance = 216.4 ± 122.5 body lengths), with larvae swimming on average 29% more distance after exposure (Fig. 3a and c). Finally, exposure to CAC did not have a significant effect on the total distance travelled (n = 19, Wilcoxon test, p-value = 0.343; baseline distance = 182.2 ± 69.4 body lengths, exposure distance = 210.3 ± 102.1 body lengths; Fig. 3a and d).

Classical conditioning and subsequent cue exposure did not have a significant effect on the swimming activity of conditioned larvae in the learning experiment (Table S2, SFigs. 3 & 4). At control temperature, exposure to control water did not significantly change the total distance travelled by conditioned larvae (n = 29, Wilcoxon test, p-value = 0.458; baseline distance = 386.8 ± 210.9 body lengths, exposure distance = 338.9 ± 198.6 body lengths). No reduction in swimming distance was found with catfish cue exposure despite the previous conditioning with mixed catfish cue and CAC (n = 21, Wilcoxon test, p-value = 0.567). Larvae travelled 343.4 ± 236.8 body lengths during the baseline period and 377.6 ± 226.2 body lengths during the exposure period. Similar results were found at elevated temperature, as distance travelled by conditioned larvae exposed to control water did not change significantly (n = 24, Wilcoxon test, p-value = 0.690; baseline distance = 247.4 ± 160.7 body lengths, exposure distance = 257.1 ± 163.5 body lengths) neither did the distance travelled by conditioned larvae exposed to catfish cue (n = 23, Wilcoxon test, p-value = 0.155; baseline distance = 269.7 ± 208.2 body lengths, exposure distance = 335.4 ± 218.5 body lengths). We made sure that a number of other factors did not significantly influence routine locomotory behaviour during the baseline activity period of both experiments, such as experimental chamber position (Kruskal–Wallis test, p-value = 0.725), holding aquarium tank (Kruskal–Wallis test, p-value = 0.912), or the cue administered during baseline period (Kruskal–Wallis test, p-value = 0.478). Finally, we also made sure well position and type of cue were not covariant (Pearson’s χ^2^ test, p-value = 0.570). Body length was found non-significantly different among thermal groups, although barely (3.4 ± 0.3 mm, Wilcoxon rank sum test, p-value = 0.067), with larvae reared at elevated temperature tending to be shorter than control larvae (SFig 5).

### Molecular responses to olfactory cues

Being reared at elevated temperature resulted in eight genes being differentially expressed compared to control temperature, in larvae not exposed to any olfactory cues. All those genes were upregulated (Table S5). Among them, three genes, *GH3 domain containing*, *mitochondrial ribosomal protein S9* and *threonyl-tRNA synthetase 2, mitochondrial* (*ghdc*, *mrps9* and *tars2*), are involved in peptide synthesis, while the gene *protocadherin 2 alpha b2* (*pcdh2ab2*) codes for a subunit of protocadherin protein, that is involved in neuronal development.

At control temperature, exposure to cues resulted in no gene being differentially expressed in whole zebrafish larvae exposed to CAC, and only in one upregulated gene, *pcdh2ab2*, in larvae exposed to catfish cue (Table S6). At elevated temperature, the *mitochondrial ribosomal protein S6* (*mrps6*; Table S7) only was downregulated in larvae exposed to CAC. However, a much larger response with 740 differentially expressed genes (Table S6) was found for catfish cue at elevated temperature, with a variety of altered key functions, such as ATP metabolism processes, protein synthesis processes, cell signalling and neurotransmission.

Among the enriched processes in larvae that developed at elevated temperature when exposed to catfish cue, cellular organisation and localization were upregulated (Table S8). Specifically, a large majority of genes involved in GTPase activity were over-expressed, enriching small GTPase mediated signal transduction and GTPase regulator activity (Table S8). Proteins encoded by these genes were either involved in the activation of GTPases or were GTPases themselves involved in signal transduction, such as Rho small GTPases or Rab protein (*rab8a)* and other types of G-protein, such as the alpha activating activity polypeptide, olfactory type (*gnal*). Furthermore, palmitoylation of proteins was also upregulated, enriching lipoprotein metabolism (Table S8), due to upregulation of genes such as *zdhhc12b* and *zdhhc5b,* coding for zinc finger DHHC-type palmitoyltransferases, and *golga7* and *golga7ba,* coding for golgins 7A proteins part of the palmitoyltransferase complex (Table S9).

**Figure 4:**
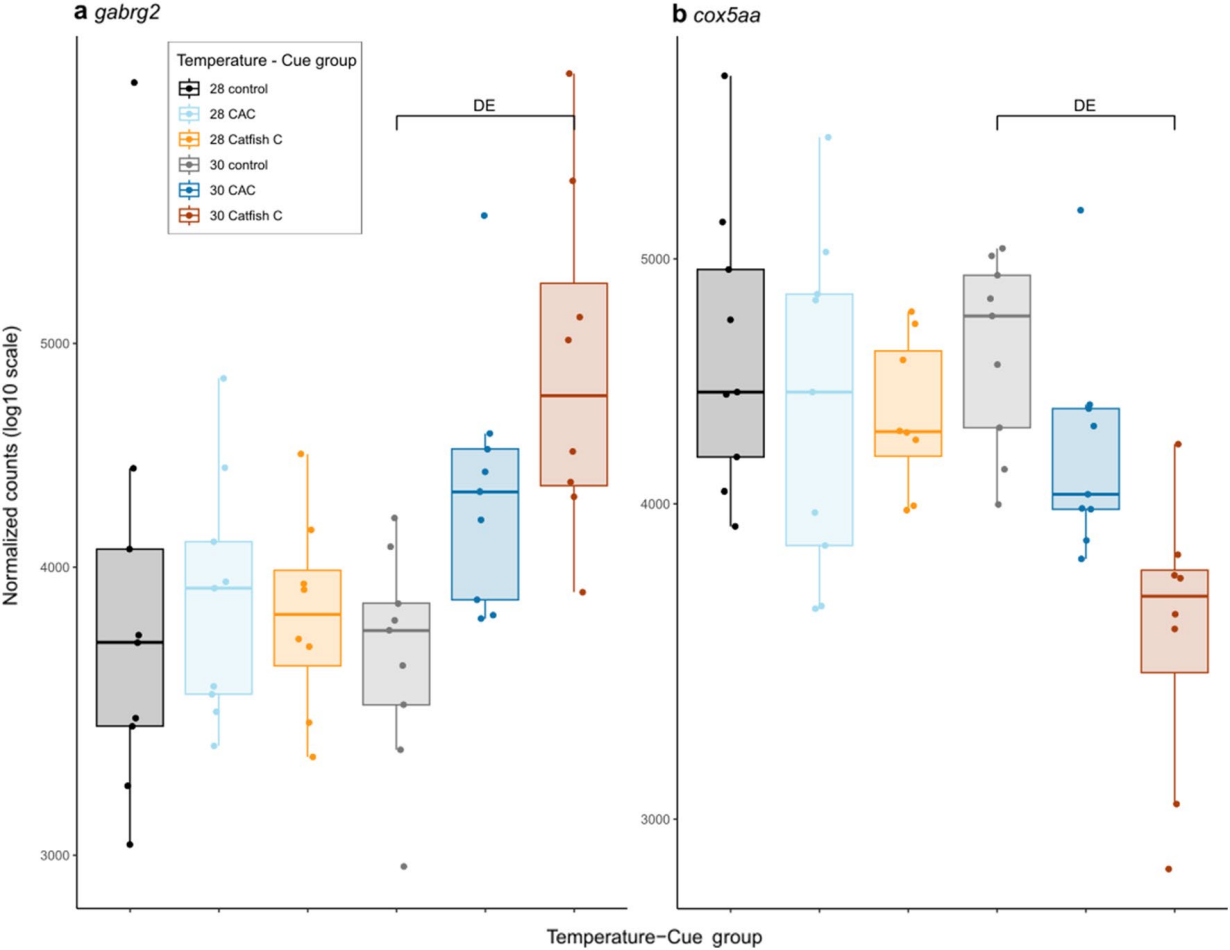
Effects of olfactory cue exposure and elevated temperature on gene expression as normalized counts (log_10_) in all temperature-cue groups for the gene *cox5aa*, a subunit of the cytochrome c oxidase (**a**) and for the gene *gabrg2*, a subunit of GABA_A_ receptor (**b**); larvae were grouped as a function of the temperature they were reared at (control, 28 or elevated, 30) and the cue they were exposed to (control, C; Conspecifics Alarm Cue, CAC; catfish cue, Catfish C); each dot represents an individual; “DE” shows which pairwise comparison revealed this gene as differentially expressed.

Finally, genes involved in neuronal development processes were upregulated (Table S8) when larvae responded to catfish cue at elevated temperature, notably through the differential expression of genes involved in axon guidance, in particular of olfactory sensory neurons (*ntn1a* and *unc5b*), contactin-associated genes (*cntnap2a* and *cntnap5a*) involved in organization of myelinated axons as well as axon growth associated genes of the semaphorin-plexin pathway (*sema4ab, plxna1a* and *plxna2)*. Additionally, genes involved in synaptic transmission were over-expressed upon exposure to catfish cue at elevated temperature. These genes belong to calcium, sodium and potassium voltage-gated ion channels (Table S9), leading to functional enrichments in metal ion binding and voltage-gated potassium channel activity (Table S8). Furthermore, γ-aminobutyric acid (GABA) receptor genes (*gabrg2*, *gabrg3* and *gabrz*; Fig. 4a; Table S9) were also upregulated.

We also found many energy-related processes altered (Table S9), such as proton transmembrane transport, mitochondrial electron transport and organization and mitochondrial ATP synthesis. This is due to the downregulation of genes involved in respiratory complexes of the electron chain (Table S8) including the NADH:ubiquinone oxidoreductase, the ubiquinol-cytochrome c reductase, the cytochrome c oxidase (Fig. 4b) and the ATP synthase (Table S8). Protein translation and homeostasis also exhibited reduced transcription upon exposure to catfish cue at elevated temperature. For example, several processes within protein synthesis were altered, including seryl-tRNA aminoacylation, rRNA binding and structural constituents of ribosome (Table S8). The same was found for protein folding, a post-translational mechanism, as two genes coding subunits of the chaperonin containing TCP1 protein (*cct2* and *cct4*) were downregulated (Table S9).

In the learning experiment, no differentially expressed genes were found in conditioned zebrafish exposed to catfish cue regardless of temperature.

## Discussion

In this study we explored how temperature, specifically predicted near-future warming, affects the innate olfactory response in zebrafish larvae and the underlying molecular drivers. We found that exposure to elevated temperature during embryogenesis until seven days post fertilization (dpf) decreased routine swimming distance and increased expression of genes involved in peptide synthesis and neuron development. However, unexpectedly, CAC did not provoke a reduction in swimming activity, no matter the rearing temperature, despite elevated temperature mediating a behavioural and a large molecular response upon exposure to non-alarming catfish cue. Finally, we found that conditioned larvae did not react to catfish cue in a way consistent with associative learning neither behaviourally nor molecularly.

Elevated temperature during embryogenesis resulted in a reduction in swimming distance in zebrafish larvae. As previous findings found no swimming alteration when seven dpf zebrafish reared at control temperature were suddenly exposed to 30 °C^39^ and that zebrafish swimming capability was found to be comparable within the range of 26–30 °C^40^, our results suggest that reduced swimming activity is an effect of developmental thermal exposure. This is consistent with reports of swimming performance being influenced by developmental temperature in zebrafish juveniles tested at a common temperature^41^. Reduced swimming could be a behavioural trade-off caused by higher energy costs of maintaining homeostasis at 30 °C, as the thermal performance curve dogma predicts^42^. Concurrently, elevated temperature triggered an over-expression of genes involved in neuron development and protein synthesis. In particular, *pcdh2ab2*, which codes for a neuronal cell-surface protocadherin expressed throughout the developing nervous system^43,44^, is upregulated in larvae at elevated temperature. Similarly, genes such as an acid-amino acid ligase (*gdhc)*, a ribosome constituent* (mrps9)* and *tars2,* which codes for an enzyme that performs the first step of translation, are found at elevated levels in elevated temperature. Upregulation of genes involved in peptide production and brain development at elevated temperature is consistent with the acceleration of metabolism and overall development due to high temperature^45^. Moreover, a 2 °C elevation was also reported to increase larval expression of growth hormone and insulin-like growth factor genes in other fish species^46^. Therefore, our results support that larvae exposed to 30 °C during embryogenesis had a faster development regarding molecular patterns, than larvae reared at control temperature.

While elevated temperature elicited a behavioural change, neither of the cues provoked freezing behaviour at control temperature. A complete suppression of locomotory movement has been previously reported in response to alarming olfactory substances in zebrafish^8,26,47^, which is likely a survival strategy to remain hidden from a predator. Therefore an absence of behavioural and molecular response to CAC is contrary to our expectations, as injured conspecific cues have been shown to reduce mobility of larvae from five to 24 dpf^26,48^. One of the possible explanations for the lack of freezing behaviour in our samples is that larvae could have responded with behaviours other than freezing, which however, were not measured here. Indeed, freezing is not the only possible behavioural response to alarm cues and zebrafish has also been shown to react to alarming cues by bottom swimming or escaping the area^25^. Alternatively, the concentration of the cue could have been under a threshold for a behavioural (and molecular) response to occur, despite the fact that we followed the methodology of Lucon-Xiccato et al.^26^, as 7 dpf larvae are smaller than 12 dpf ones. Indeed, previous research found that intensity of the alarm response is dose-dependent^25^.

Catfish cue was used as a “neutral non-alarming cue” since the catfish (*Pangasianodon hypophthalmus*) is not a known alarming presence to lab-reared zebrafish and its smell has not been found to trigger a freezing response^26^, therefore unchanged activity at control temperature following catfish cue exposure was consistent with our expectations. Interestingly, exposure to catfish cue elicited expression changes of *pcdh2ab2* at control temperature. In seahorses (*Hippocampus erectus*), *pcdh2ab2* is also changed in expression levels when faced with the visual and olfactory signal of a paired mate^49^. As *pcdh2ab2* is involved in neuron development, it could play a role in the neuronal alterations in the context of odour detecting and olfactory memory. In our case, since this gene was not also upregulated in larvae exposed to CAC, the reason for upregulation of this gene upon smelling catfish cue might be due to the novelty of this specific cue in the larvae’s environment. Unlike CAC, which was prepared with larvae of the same school, catfish cue was never introduced before and this was the first exposure to this new smell for the larvae. Interestingly, exposure to elevated temperature caused an increase in swimming activity in response to catfish cue. Previous occurrences of increased locomotory activity were reported in zebrafish as a response to a variety of odours, such as food, social cues or decay odours^50,51^, as well as amino acids in larvae as early as 4 dpf^52^. Here, increased activity in the presence of the novel catfish cue at elevated temperature could be due to zebrafish larvae displaying an aversive response similar to decay odour avoidance^53^, with catfish cue possibly containing dead skin compounds. Since older larvae do not react to catfish odour alone at control temperature^26^, it is unlikely that the reason larvae only responded to this cue at elevated temperature is solely accelerated development. Rather, temperature-induced stress may increase the fish sensitivity to olfactory stimulation and therefore decrease the threshold for a behavioural response to occur.

Elevated temperature mediated a large transcriptional response to the novel smell of catfish cue exposure. Processes involved in cellular signalling, cell organisation and localization were upregulated, including genes involved in GTPase activity essential for signal transduction, particularly in G-proteins signalling pathways. Small G-protein coding genes of the Rho family participate in cell shaping^54^, while genes coding for Rab proteins like *rab8a* are involved in the elongation of sensory cilia^55^, which are cell organelles harbouring olfactory receptors^56^. Interestingly, the olfactory specific G-protein gene *gnal* was upregulated as well, which codes for an olfactory specific G-protein expressed in the ciliated olfactory sensory neurons of zebrafish and is involved in olfactory map refinement in the olfactory epithelium^57,58^. Similarly, odorant exposure in mice leads to transcriptional changes in cell signalling and G-protein-coupled receptor activity in olfactory sensory neurons^59^ and novel odour in particular provokes expression changes in cytokine-mediated cell signalling genes in rats^60,61^. Similar processes therefore seem to occur in fish after detecting a novel odour in the environment at high temperature, showing that there is a change in signalling activity possibly involved in neuronal olfactory circuitry modifications in response to novel odours.

Together with cell signalling, genes involved in neuronal development were also upregulated in response to catfish cue at elevated temperature. In particular, *ntn1a* and *unc5b* participate in sensory axon targeting in the olfactory bulb^62,63^. Moreover, over-expression of contactin-associated genes, such as *cntnap2a*, required for proper organization of myelinated axons^64^, suggests that the cellular organization of neurons is modified after catfish cue exposure. Finally, genes of the semaphorin-plexin pathway participate in axon growth, notably of neurons of the olfactory system^65,66^, further supporting the hypothesis that detection of novel odours at high temperature triggers neuronal growth. Other upregulated genes participated in general neurotransmission, such as voltage-dependant ion channels and neurotransmitter receptors like GABA_A_ receptors. The reason for such a strong response to catfish cue at elevated temperature only might again lie in thermally-induced accelerated development, in particular of the nervous system, with larvae that developed at elevated temperature potentially having more neurons and/or synapses at elevated temperature compared to same age larvae reared at control conditions^45^. Along with accelerated development, warming during the larval stage could also provoke changes to neuronal circuitry resulting in altered olfactory detection. This is the case of honeybees, for example, in which the olfactory-input region of the brain is altered due to differences in developmental temperature^67^. The upregulation of axon guidance genes in particular supports both hypotheses, as it would allow more axons to project into the olfactory bulb. Whether through accelerated development, altered olfaction neural circuitry or a combination of both, temperature has therefore been found here to strongly impact novel odour response in fish at the molecular level, suggesting neuromolecular changes in a future warming world.

On the contrary, different genes coding for components of the electron transport chain were downregulated. Downregulation of electron transport chain genes is expected to reduce mitochondrial respiration, which could relate to hypometabolism under heat stress, as previously seen in mitochondria of other fishes at elevated temperature^68,69^. Genes involved in protein synthesis were also downregulated, which further indicates the repression of several metabolic processes due to temperature-induced increased metabolism. A possible explanation to why this type of response to catfish cue is only observed at elevated temperature could be that detecting new odours while maintaining homeostasis at high temperature requires additional energy. This therefore creates a trade-off between expensive basal processes, such as protein production, and novel odour response. Since protein synthesis is one of the most expensive cellular processes, it is also one of the first ones that is repressed^70^, here to redirect energy to the odour response. Consequently, when a novel odour is detected, energetic reserves would shift towards olfactory responsive functions, possibly leading to energy resource depletion and long-term detrimental effects. Although this large metabolic repression is observed after exposure to catfish cue, at elevated temperature CAC also triggered downregulation of the ribosomal protein coding *mrps6* gene, which could indicate that energetic trade-offs are necessary to respond to several types of olfactory cue at elevated temperature. Overall, our results therefore show how future-predicted temperature exposure during development mediates increased expression of genes involved in cell communication, neuron functioning and development, at the cost however of a repression of key metabolic processes following an otherwise non-noxious environmental stimulus that is the catfish cue. Such energy reallocation needed during processing an environmental signal might negatively affect basic processes necessary to development and growth in the long run, when faced with future global warming conditions.

Conditioning did not result in any behavioural or molecular response, with conditioned larvae not exhibiting freezing behaviour or gene expression changes following catfish cue exposure regardless of temperature. Older 24 days post-fertilization (dpf) larvae respond to catfish cue by freezing after conditioning with CAC^26^, and some larvae of similar age to ours also demonstrate associative learning through classical conditioning, although to visual stimuli and not olfactory cues^71^. Despite evidence of successful learning at seven dpf, the possibility that larvae were not developed enough for robust learning cannot be discarded, as the target performance in previous associate learning studies were modest and not always consistent across all larvae^71,72^. Regarding the absence of gene expression changes upon smelling catfish cue in conditioned larvae, these findings reinforce our hypothesis of the novel odour stress response at high temperature. Contrary to the innate experiment, where the large molecular response to the catfish cue was seen when larvae had their first exposure to it, in the conditioning experiment larvae were tested for a response to the catfish cue based on their previous experience with no large molecular reprogramming. In summary, our findings show an effect of future-predicted thermal conditions on developing zebrafish, causing changes in swimming during routine activity and, as larvae encounter a new olfactory stimulus, largely altering gene expression processes in the nervous system, making environmental sensing energetically costly.

### Supplementary Information

Supplementary Figures.

Supplementary Tables.

## Data Availability

The raw sequencing data can be found in BioProject PRJNA974200. The reviewer link to the data is: https://dataview.ncbi.nlm.nih.gov/object/PRJNA974200?reviewer=n5uqu49k1qf7jj3ljqijee7oj8.
