# Supplementary material for: Warming affects routine swimming activity and novel odour response in larval zebrafish
Source: Sci Rep. 2023 Nov 29;13:21075. doi: 10.1038/s41598-023-48287-y (PMC10687225; doi:10.1038/s41598-023-48287-y)
Supplement: Supplementary file 1 — Supplementary Figures. [file 41598_2023_48287_MOESM1_ESM.pdf]

Warming affects routine swimming activity and novel odour response in larval zebrafish

Jade M. SOURISSE[1], Lucrezia C. BONZI[1], Julie SEMMELHACK[2], and Celia SCHUNTER[1]*

[1] The Swire Institute of Marine Science, School of Biological Sciences, The University of Hong Kong, Pokfulam Road, Hong Kong SAR

[2] The division of Life Science, Department of Chemical and Biological Engineering, The Hong Kong University of Science and Technology, Clearwater Bay, Kowloon, Hong Kong SAR

Supplementary Figures:

**Name**	**Title**
Supplementary Figure 1	Measured temperature (°C) regimes in each experimental group (control or elevated) throughout the experiments; stars (***) indicate the significant difference between the mean values
Supplementary Figure 2	Total distance (body length) travelled by the larvae reared in control temperature, before cue exposure (baseline) and after cue exposure (exposure) for each cue group: control water (a), catfish cue (b) or CAC (c); black dots linked by full lines represent paired individual comparisons of before and after the cue exposure; "NS." stands for "non-significant" and illustrate the absence of a statistical difference between the mean values
Supplementary Figure 3	Total distance (body length) travelled by the conditioned larvae reared in control temperature, before (baseline) and after cue exposure (exposure) to control water (a) or catfish cue (b); black dots linked by full lines represent paired individual comparisons of before and after the cue exposure; "NS." stands for "non-significant" and illustrate the absence of a statistical difference between the mean values
Supplementary Figure 4	Total distance (body length) travelled by the conditioned larvae reared in elevated temperature, before (baseline) and after cue exposure (exposure) to control water (a) or catfish cue (b); black dots linked by full lines represent paired individual comparisons of before and after the cue exposure; "NS." stands for "non-significant" and illustrate the absence of a statistical difference between the mean values
Supplementary Figure 5	Larval length (mm) density according to their experimental group, control (blue) or elevated (red) temperature; dotted lines represent the mean value in each group

Supplementary Figure 1:

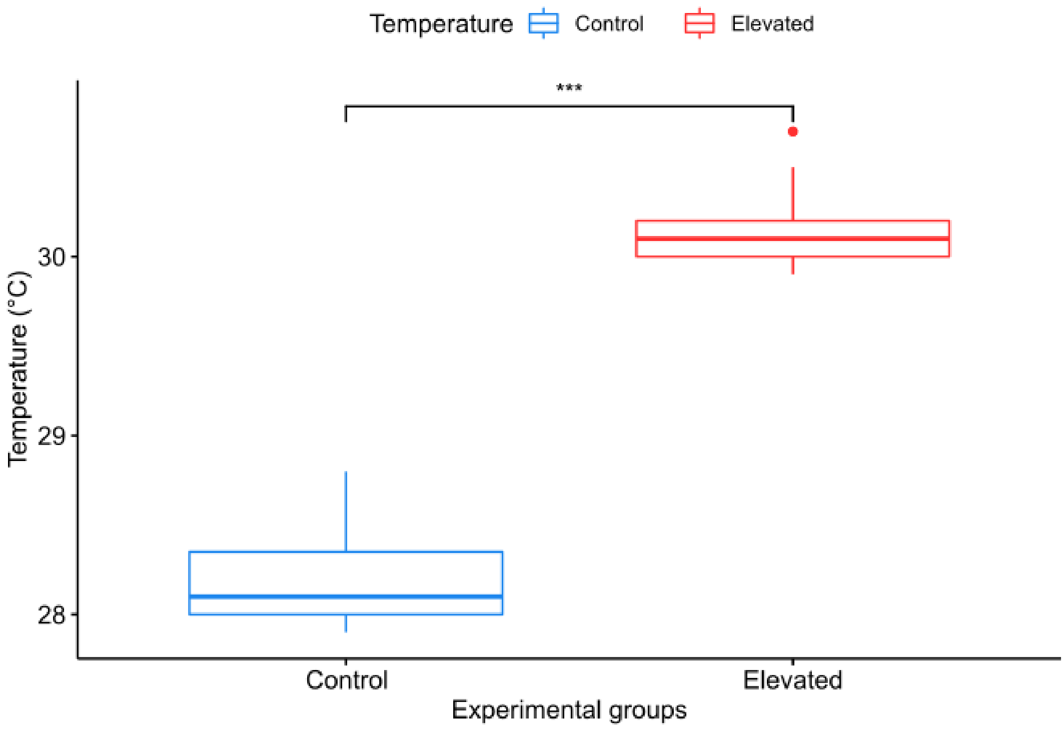

Supplementary Figure 1: Measured temperature (°C) regimes in each experimental group (control or elevated) throughout the experiments; stars (***) indicate the significant difference between the mean values

Supplementary Figure 2:

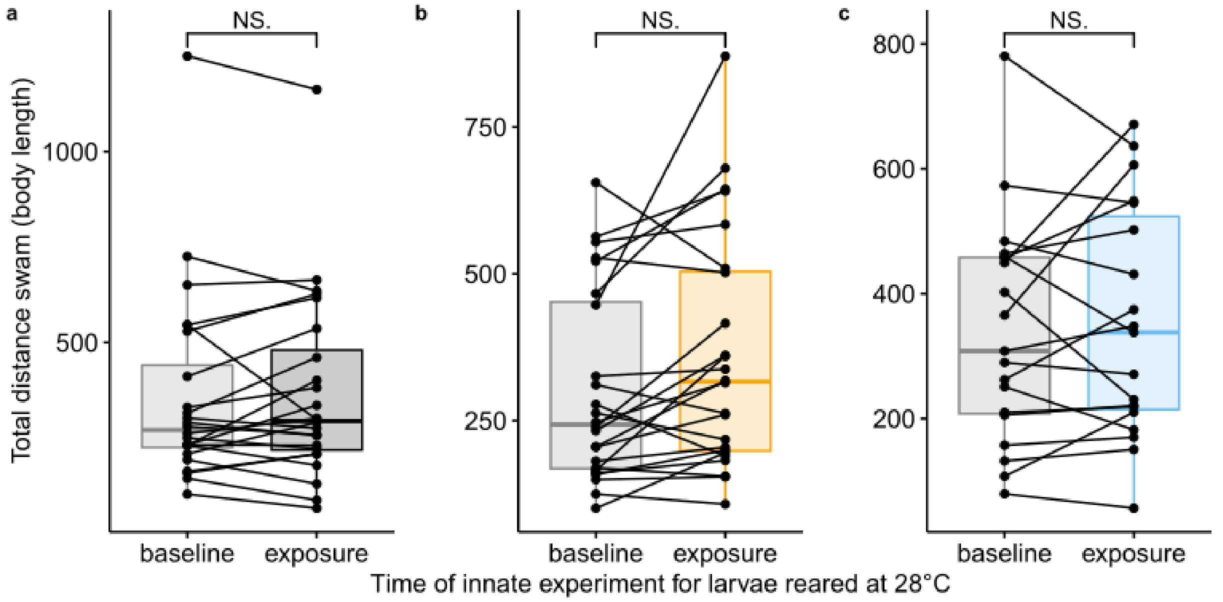

Supplementary Figure 2: Total distance (body length) travelled by the larvae reared in control temperature, before cue exposure (baseline) and after cue exposure (exposure) for each cue group: control water (a), catfish cue (b) or CAC (c); black dots linked by full lines represent paired individual comparisons of before and after the cue exposure; "NS." stands for "non-significant" and illustrate the absence of a statistical difference between the mean values

Supplementary Figure 3:

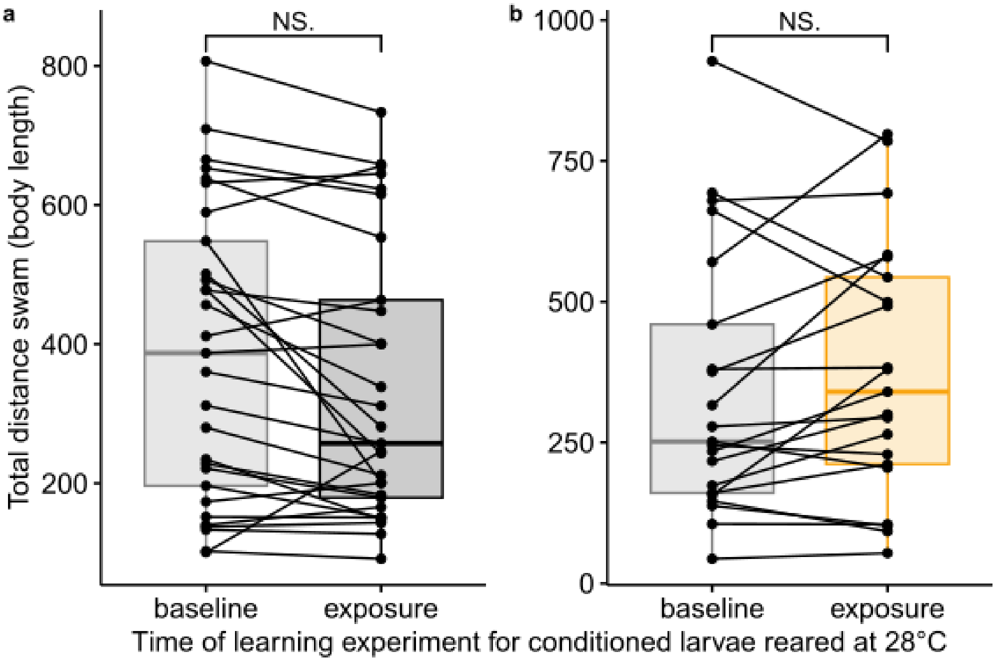

Supplementary Figure 3: Total distance (mm) travelled by the conditioned larvae reared in control temperature, before (baseline) and after cue exposure (exposure) to control water (a) or catfish cue (b); black dots linked by full lines represent paired individual comparisons of before and after the cue exposure; "NS." stands for "non-significant" and illustrate the absence of a statistical difference between the mean values

Supplementary Figure 4:

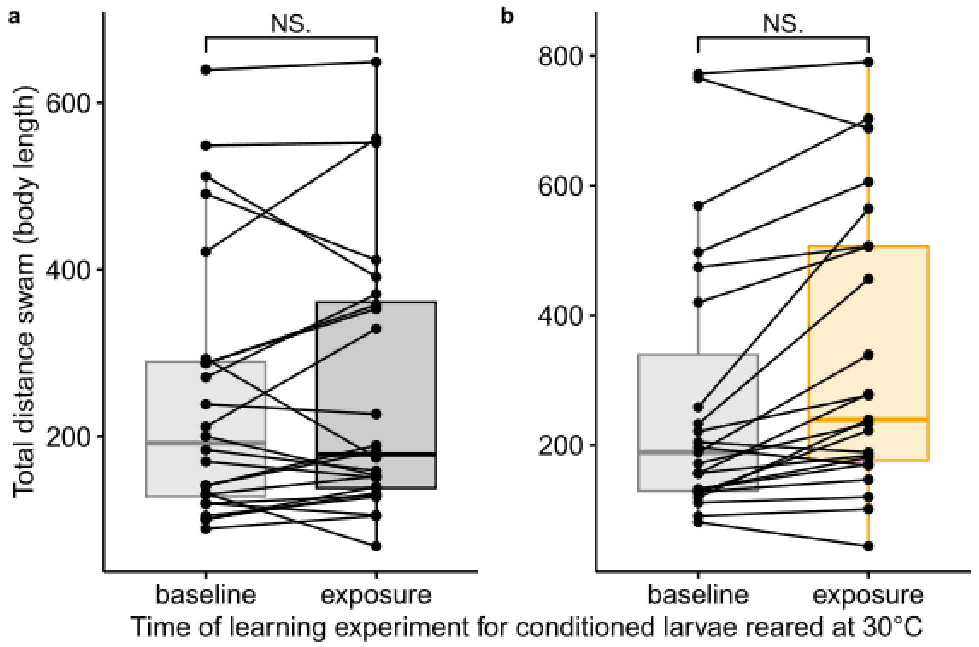

Supplementary Figure 4: Total distance (body length) travelled by the conditioned larvae reared in elevated temperature, before (baseline) and after cue exposure (exposure) to control water (a) or catfish cue (b); black dots linked by full lines represent paired individual comparisons of before and after the cue exposure; "NS." stands for "non-significant" and illustrate the absence of a statistical difference between the mean values

Supplementary Figure 5:

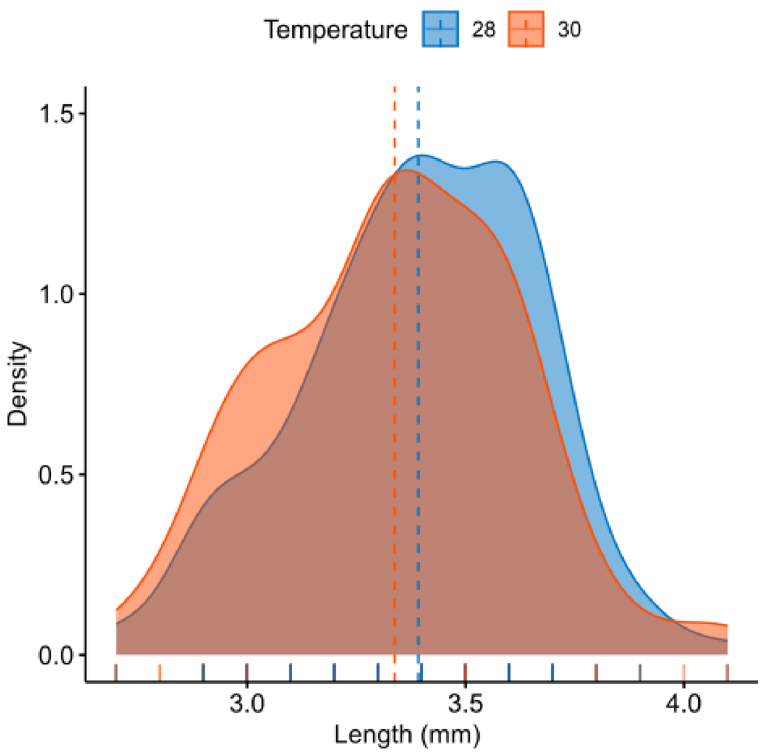

Supplementary Figure 5: Larval length (mm) density according to their experimental group, control (blue) or elevated (red) temperature; dotted lines represent the mean value in each group
